# Fabrication of CL-20/HMX Cocrystal@Melamine–Formaldehyde Resin Core–Shell Composites Featuring Enhanced Thermal and Safety Performance via In Situ Polymerization

**DOI:** 10.3390/ijms23126710

**Published:** 2022-06-16

**Authors:** Binghui Duan, Xianming Lu, Hongchang Mo, Bojun Tan, Bozhou Wang, Ning Liu

**Affiliations:** 1 Xi’an Modern Chemistry Research Institute, Xi’an 710065, China; duanbinghui@126.com (B.D.); luxianming1220@126.com (X.L.); hongchangmo@163.com (H.M.); tanbj204@163.com (B.T.); 2 State Key Laboratory of Fluorine & Nitrogen Chemicals, Xi’an 710065, China

**Keywords:** CL-20/HMX cocrystal, in situ polymerization, core–shell structure, desensitization, thermal stability

## Abstract

Safety concerns remain a bottleneck for the application of 2,4,6,8,10,12-hexanitro- 2,4,6,8,10,12-hexaazaisowurtzitane (CL-20)/1,3,5,7-tetranitro-1,3,5,7-tetrazacyclooctane (HMX) cocrystal. Melamine–formaldehyde (MF) resin was chosen to fabricate CL-20/HMX cocrystal-based core–shell composites (CH@MF composites) via a facile in situ polymerization method. The resulted CH@MF composites were comprehensively characterized, and a compact core–shell structure was confirmed. The effects of the shell content on the properties of the composites were explored as well. As a result, we found that, except for CH@MF–2 with a 1% shell content, the increase in shell content led to a rougher surface morphology and more close-packed structure. The thermal decomposition peak temperature improved by 5.3 °C for the cocrystal enabled in 1.0 wt% MF resin. Regarding the sensitivity, the CH@MF composites exhibited a significantly reduced impact and friction sensitivity with negligible energy loss compared with the raw cocrystal and physical mixtures due to the cushioning and insulation effects of the MF coating. The formation mechanism of the core–shell micro-composites was further clarified. Overall, this work provides a green, facile and industrially potential strategy for the desensitization of energetic cocrystals. The CH@MF composites with high thermal stability and low sensitivity are promising to be applied in propellants and polymer-bonded explosive (PBX) formulations.

## 1. Introduction

**Figure 1:**
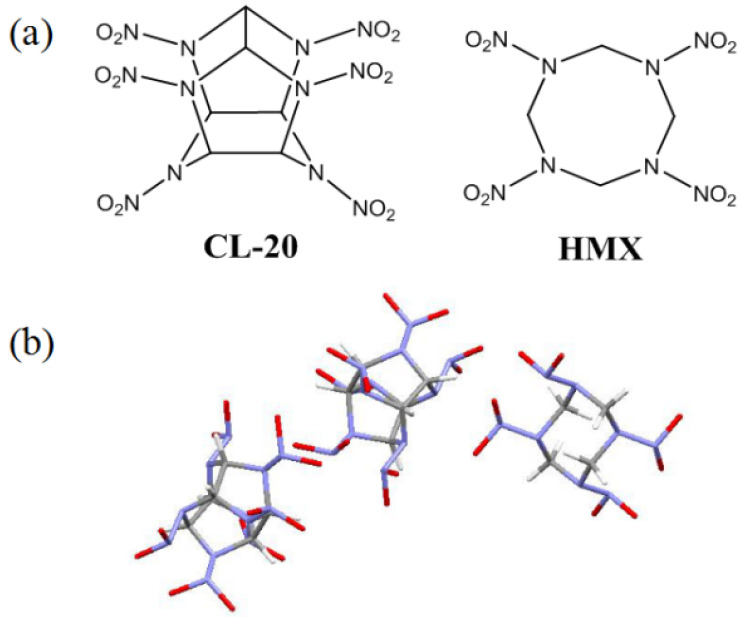
The molecular structures of (**a**) CL-20 and HMX; (**b**) 2:1 CL-20:HMX cocrystals.

Over the last decade, advanced and safe energetics witnessed the rapid development of energetic cocrystallization. Cocrystal is a kind of crystalline substance composed of two or more neutral molecules, which has been proved to effectively release the contradiction of safety and power of energetic materials (EMs) [1,2,3,4,5]. 2,4,6,8,10,12-Hexanitro-2,4,6,8,10,12-hexaazaisowurtzitane (CL-20) was reported to cocrystallize with less sensitive EMs such as 2,4,6-trinitrotoluene (TNT) [6], 1,3,5,7-tetranitro-1,3,5,7-tetrazacyclooctane (HMX) [7], benzotrifuroxan (BTF) [8], 2,5-dinitrotoluene (DNT) [9], 1-methyl-3,4,5-trinitropyrazole (MTNP) [10], and 1-methyl-3,5-dinitro-1,2,4-triazole (MDNT) [11]. Therein, CL-20/HMX cocrystal stood out for its superior performance. Matzger’s group [7] found that this cocrystal has a comparable detonation velocity to CL-20 and a similar impact sensitivity level with pure HMX. The chemical structures of CL-20 and HMX and the molecular structure of CL-20/HMX cocrystal are shown in Figure 1. The spray drying, self-assembly and resonant acoustic mixing methods have pushed the scaled-up production of CL-20/HMX cocrystal to kilograms creatively [12,13,14]. However, the application of CL-20/HMX cocrystal is faced with two dilemmas: one is that although cocrystallization lowers its sensitivity to some extent, the safety of CL-20/HMX cocrystal is still far from satisfactory in practical formula; the other is that certain amounts of additives, such as graphene, paraffin, polymer or other insensitive additives, need to be added in consideration of the desensitization, but the traditional mechanical mixing method suffers from low efficiency. Studies show that explosion under impact is likely to occur on the surface of explosive particles first; thus, the surface coating may be efficient to reduce the sensitivity of EMs [15,16,17,18,19,20].

The core–shell technology of EMs is mainly derived from coating technology, which can be traced back to the microencapsulation process in the field of medicine and food [21,22,23]. Microencapsulation is a type of technical means that the fine particles of substance (i.e., solid, liquid or gas) are coated in a thin shell of polymer membrane [24,25,26]. The core particles are separated from the external environment through the shell, meanwhile changing their energy release. The EMs with a core–shell structure feature three pronounced advantages: close contact between components; combined functionalities of core and shell; improved overall performance [27,28,29,30]. Close contact between components is the main characteristic of core–shell EMs (CSE) that distinguishes them from physical mixed composites. 

There are several techniques to prepare CSEs [31,32,33,34,35]. The water suspension method is the most common practice to prepare CSEs due to the moderate processing conditions and versatility for most coating systems. However, the coating efficiency is not satisfactory, and the aggregation of particles has been frequently reported for small grains of explosives. The emulsion method is sometimes used to fabricate spherical core–shell composites in industrial processes. The key is to find a proper demulsifier during the process. One drawback is that various chemical additives are added in the emulsion process, thus increasing the risk of chemical incompatibility among the components. An in situ polymerization method has been extensively applied to microencapsulation and surface coating. The process involves monomer adhesion on the core surface and subsequent polymerization, which can facilitate a higher coverage and adhesion force than a simple physical mixing process. The desirable stability and mechanical performance could be achieved with the low addition of shell materials. Thus far, there are almost twenty kinds of candidates accessible as shell materials. Therein, the use of melamine–formaldehyde (MF) resins has attracted many researchers in that MF prepolymers possess high reactivity, short reaction times and small amounts of polymer needed to construct a complete shell [36]. Yang et al. prepared a series of CSEs via in situ polymerization of MF resin on the surface of explosive crystals [37]. It was found that the energetic cores were sufficiently covered by the MF resin with a 3% shell content.

On the basis of the above work, core–shell CL-20/HMX cocrystal@MF resin (CH@MF) microparticles were prepared based on an in situ polymerization strategy in this study. As far as we know, this is the first report to apply core–shell technology to energetic cocrystal handling. The morphology and core–shell structure were first examined, and then the surface properties, thermal behavior, sensitivity and detonation performance of the composites were comprehensively investigated. The formation mechanism of CH@MF composite was explored as well. This work aimed to provide a better understanding of core–shell formation mechanism and promote the further application of cocrystal energetics in propellants and PBX formulations.

## 2. Results

### 2.1. Morphologies

**Figure 2:**
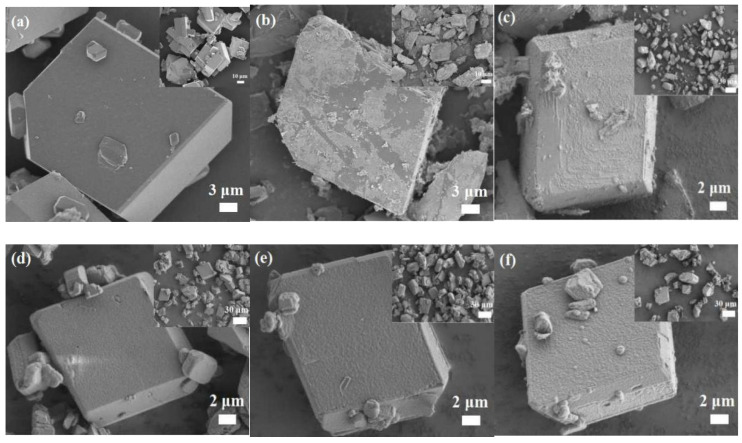
SEM images of (**a**) neat CL-20/HMX cocrystal and (**b**) CH@MF–1 (0.5%); (**c**) CH@MF–2 (1.0%); (**d**) CH@MF–3 (1.6%); (**e**) CH@MF–4 (2.8%); (**f**) CH@MF–5 (3.5%). Corresponding images with low magnification are inserted.

For explosives, the morphology, size and arrangement states play an important role in many military applications. The surface morphologies of CL-20/HMX cocrystal and CH@MF composites were examined through SEM measurements and are presented in Figure 2. One can see from Figure 2a that the CL-20/HMX cocrystals exhibited neat and smooth surfaces with a cuboid shape. Several small particles were attached on the surface, and the crystal size ranged from 5 to 30 μm. A certain amount of MF resin was designated to coat the CL-20/HMX cocrystal via in situ polymerization. The core-etching method was applied to identify the content of the CL-20/HMX cocrystal in the composite; then, the content of the MF resin could be determined (w_MF_ = 100% − w_CH_). Herein, the micro-composites were stirred vigorously for 30 min in excessive acetone to dissolve the CL-20/HMX cocrystal absolutely; then, the suspensions were filtered with a 0.45 μm polytetrafluoroethylene filter to decide the cocrystal content, and the residual solids were MF resins. The effect of the shell content on the morphology was studied and the amounts of MF resin were 0.5%, 1.0%, 1.6%, 2.8% and 3.5% for CH@MF–1, CH@MF–2, CH@MF–3, CH@MF–4 and CH@MF–5, respectively. After MF modification, the shape of the particles exhibited no noticeable change. It could be found that the surface of the CH@MF composites became rough owing to the existence of small MF particles. Nevertheless, the grain MF resin did not form an entire shell on the core surface of CH@MF–1 (Figure 2b), resulting in a limited coating effect. For CH@MF–2 (Figure 2c), the polymeric MF resin covered the surface of the CL-20/HMX cocrystal basically with some granular deposition. When it comes to CH@MF–3, as shown in Figure 2d, a compact layer of MF resin was obviously found on the surface of the cocrystal with full-scale coverage. Meanwhile, the edges of the CL-20/HMX cocrystal became blunt after a sufficient coating. In Figure 2e, the coating layer of the CH@MF–4 composite displays a rough, thick and intact morphology. In addition, when the shell content of the MF resin increased to 5.7 wt%, the CH@MF–5 composite (Figure 2f) exhibited a rougher and thicker morphology. Some agglomerations were observed on the surface, implying that the addition of shell should be controlled moderately, considering the coating efficiency and energy loss comprehensively. One should note that CL-20/HMX cocrystal will gradually crack when exposed to an electron beam. After coating, the MF shell behaved like an armature to protect the cocrystal from the electron beam and almost no cracks could be observed on the surface of the CH@MF composites.

**Figure 3:**
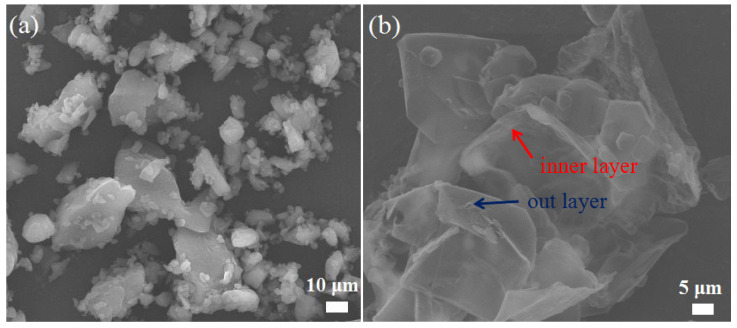
SEM images of the physical mixture of CL-20/HMX cocrystal and MF resin (**a**); MF resin shell after etching of the cocrystal core of CH@MF–3 (**b**).

In addition, the physical mixture of the CL-20/HMX cocrystal and MF resin as well as the MF resin after the etching of the cocrystal core of CH@MF–3 were tested. As shown in Figure 3a, most of the MF resin particles agglomerated rather than deposited on the surface of the cocrystal, indicating a negligible coating effect. It can be observed from Figure 3b that after the inner cocrystal cores were dissolved by acetone, the insoluble MF shells retained the shape of the cocrystal, and the shell thickness was approximately 1~2 μm. In addition, the internal layer of the MF shell presented a similar smoothness to the surface of the core explosive, while the outer layer was tough. These works realized that the cocrystals coated with the MF resin showed the typical features of a well-developed core@shell structure.

**Figure 4:**
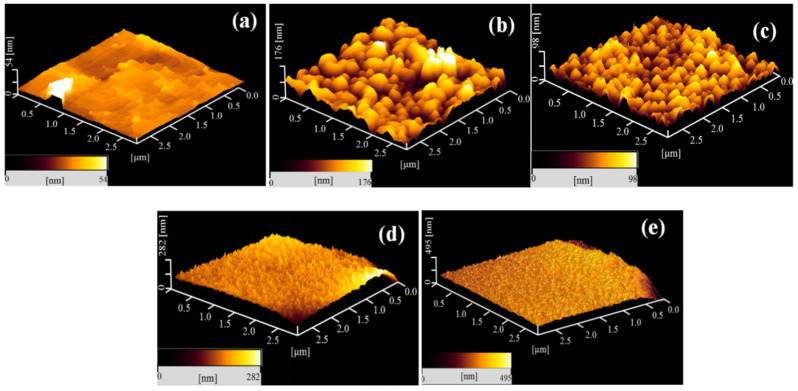
The AFM images of the CL-20/HMX cocrystal and MF coating composites: (**a**) CL-20/HMX cocrystal; (**b**) CH@MF–2; (**c**) CH@MF–3; (**d**) CH@MF–4; (**e**) CH@MF–5.

The surface morphology and roughness were further characterized based on AFM measurements. The AFM images of the CL-20/HMX cocrystal and MF coating composites with different shell contents are presented in Figure 4. The surface roughness parameters, including the mean value of roughness (R_a_), root mean square roughness (R_q_) and the maximum height value from peak to valley (R_max_) of the samples are shown in Table 1. The neat CL-20/HMX cocrystal exhibited a relatively flat and smooth surface. After MF modification, the cocrystal was tightly wrapped with grain MF particles and a rough surface was generated. With the increasing shell content, the coverage increased due to the increased frequency of occurrence and the assemblage of MF resin on the surface. The gradual increase in the frequency of the observation of these MF particles implies that the polymeric MF resin particles may deposit layer by layer on the crystal surface, contributing to an even and compact coating shell. For CH@MF–2 with a shell content of 1.0%, the surface roughness increased strikingly with R_a_ = 29.8 nm and R_q_ = 22.7 nm. It is interesting to note that the surface roughness decreased, and the MF particles arranged more evenly when the shell content increased to 1.6%, which is consistent with the morphology observed from the SEM test. This may be attributed to the fact that a more homogeneous surface deposition and higher coating efficiency could be obtained in the case that the shell content came to a specific degree, i.e., 1.6%. The surface roughness continued to increase with the rising shell content, together with a flat and close-packed surface morphology. It was deemed that the increase in surface roughness promoted the interfacial interaction between the composite and polymer matrix.

### 2.2. Structural Characterizations

**Figure 5:**
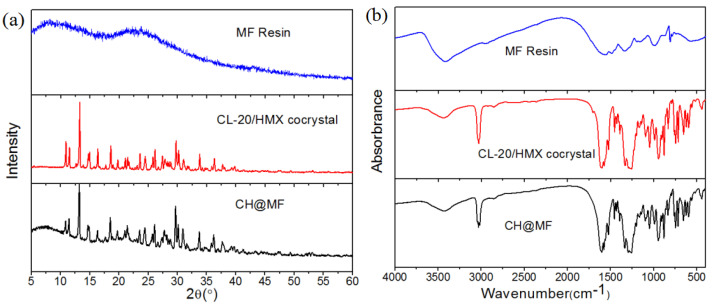
XRD (**a**) and IR (**b**) spectra of the MF resin, CL-20/HMX cocrystal and CH@MF–3 composite.

The core–shell structures and component states of the CH@MF composites were investigated by XRD patterns, IR and Raman spectra. The CH@MF–3 composite with a shell content of 1.6% was adopted as an example. It can be seen from Figure 5a that the structure of the MF resin was amorphous, while an obvious crystalline structure was found for the CL-20/HMX cocrystal with characteristic diffraction peaks (i.e., 10.92°, 13.29°, 14.83°, 18.56°, 29.78° and 30.9°) coincidental with XRD spectra in the literature [7]. The peak pattern and position of CH@MF–3 were in accordance with those of the cocrystal, proving that the surface modification of the MF resin had little impact on the cocrystal’s structure. One should note that the addition of MF resin influenced the peak intensity of the composite. For example, the peak intensity of 2θ at 10.92º, 23.53º decrease in comparison with the pure cocrystal, while 2θ at 21.39º, 31.59º increase. This may be explained by the fact that the MF resin coating on the cocrystal surface acted on the preferred orientation of the crystals. This demonstrates that the cocrystals were successfully covered by low contents of MF resin and the polymorph of the cocrystal did not change during the whole process.

Figure 5b reveals the IR spectra of the CL-20/HMX cocrystal, MF resin and CH@MF–3 composite. The assignments of main vibrational bands of IR spectra of MF resin and CL-20/HMX cocrystal are presented in Table S1. It can be seen that the cocrystal showed a strong absorption band at 3033 cm^−1^, which could be assigned to the C–H stretching vibration. The –NO_2_ symmetric stretching vibrations were observed at 1602 and 1577 cm^−1^. The characteristic peaks confirmed the cocrystal structure as referenced in the literature [7]. For the MF resin, the broad band at approximately 3427 cm^−1^ could be identified as hydroxyl, imino and amino stretching. C–N multiple stretchings in the triazine ring were observed at 1561 and 1491 cm^−1^. Characteristic triazine ring deformation at 810 cm^−1^ could also be observed. After MF coating, the IR characteristic diffractions of the cocrystal were retained. The Raman spectra study (Figure S1, Table S2) came to the same conclusion with the IR analysis. The low content of the MF resin and the overlap of the characteristic peaks of both of components may be responsible for this phenomenon.

**Figure 6:**
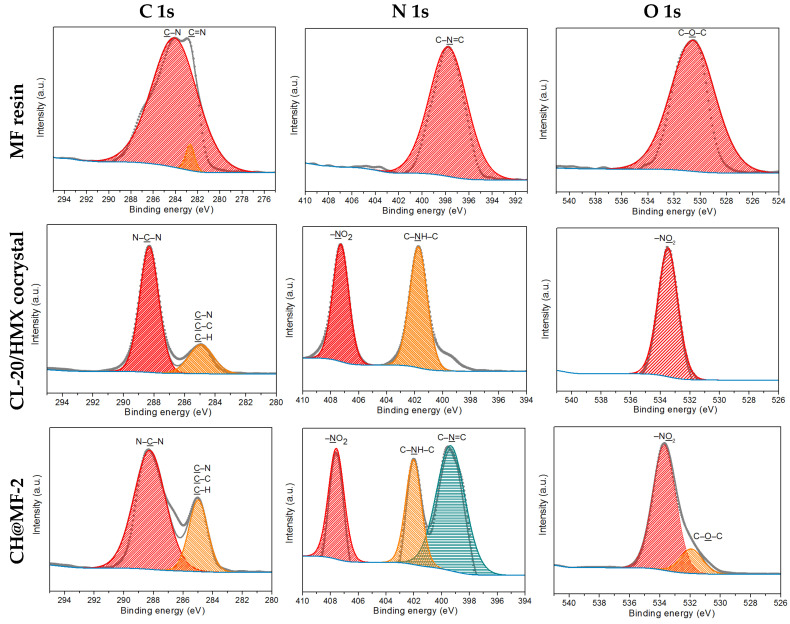

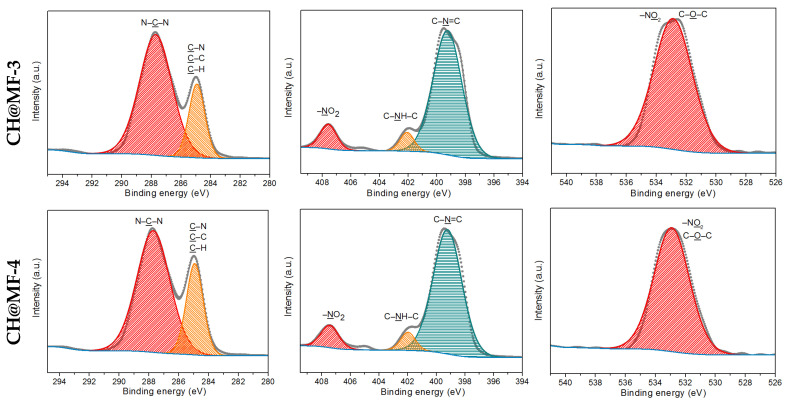
XPS spectra of the C 1s, N 1s and O 1s regions for the MF resin, CL-20/HMX cocrystal and corresponding CH@MF composites, respectively.

An XPS analysis was conducted to elucidate the surface chemical compositions of the CH@MF composites to confirm the coating efficiency, and the spectra are given in Figure 6. The surface element percent of the C 1s, N 1s and O 1s atoms in the MF resin, CL-20/HMX cocrystal, and CH@MF composites were integrated and are presented in Table 2. The C 1s peaks in the MF resin were attributed to C–N (285.3 eV) and C=N (282.7 eV). The N 1s peak that occurred at 397.8 eV could be denoted as C–N=C. The peak at 530.8 eV fitted in the O 1s region was attributed to C–O–C. For the CL-20/HMX cocrystal, two peaks were fitted in the C 1s region and assigned to C–H (or C–C, C–NO_2_) and N–C–N. The N 1s peak was composed of two peaks, which were C–NH–C at 401.7 eV and –NO_2_ at 407.3 eV. The O 1s peak centered at 533.5 eV was assigned to the –NO_2_ group. After the coating of the CL-20/HMX cocrystal with the MF resin, the basic shape of the C 1s profiles remained unchanged due to the overlap of the C–N peak in the MF resin and the cocrystal. The peaks centered at approximately 285.0 eV (ascribed to C–H, C–N and C–C) showed an increased intensity with the addition of MF resin. The basic N 1s region exhibited the sum characteristics of cocrystal and MF, with three peaks attributed to –NO_2,_ C–NH–C and C–N=C in sequence. Compared with the CL-20/HMX cocrystal, the increased shell content contributed to the decreased atomic concentrations of –NO_2_ and C–NH–C and increased atomic concentrations of C–N=C, which only belonged to MF in the XPS analysis. The C/N ratio of the CH@MF composite was between that of the cocrystal and MF, and it showed a positive correlation with the shell content. With further increase of the shell content to 2.8% for CH@MF–4, obvious improvements in the atomic concentrations of C–N and C–N=C as well as the C/N ratio was found. When it came to the O 1s profile, the featured peak of C–O–C at 531.9 eV emerged for CH@MF–2. With the further increase in the MF concentration, the O 1s profile exhibited only one broad peak at approximately 532.7 eV, as the close peaks of C–O–C and –NO_2_ appeared to be superimposed. The XPS atomic concentration of functional groups in MF, CL-20/HMX cocrystal and CH@MF composites are listed in Table S3. One can find from Table 2 and Table S3 that the nitrogen content decreased and the carbon content increased as the shell content rose, which may be ascribed to the increased polymerization degree and a relatively high carbon content in the MF resin. It follows from these results that the cocrystal surface was successfully coated by the MF resin via in situ polymerization.

### 2.3. Surface Analysis

**Figure 7:**
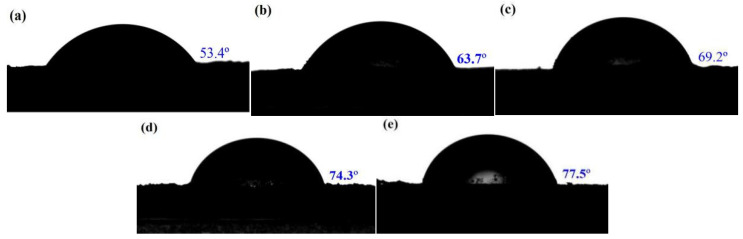
The static contact angles of (**a**) CL-20/HMX cocrystal; (**b**) CH@MF-2; (**c**) CH@MF-3; (**d**) CH@MF-4; (**e**) CH@MF-5 with test fluid of water.

Characterization of surface properties by measuring contact angles is a useful method to explore the influence of the surface coating on the interaction between the cocrystal and fluoropolymer (F_2314_) binder in a PBX matrix. The static contact angles of CL-20/HMX cocrystal and CH@MF composites with test fluid of water are presented in Figure 7. The neat CL-20/HMX cocrystal showed a low water contact angle of 53.4°. The water contact angle of the CH@MF increased in the presence of hydrophobic MF resin with nonpolarity characteristics. The water contact angle of the CH@MF composites continued to rise with the increasing shell content, suggesting a stable storage performance in atmosphere.

Based on the contact angles of the different droplets on the energetic crystals, the surface energy (*γ*) was described by Equation (1). The interfacial adhesion work (*W_a_*) was further calculated by the surface energy components of the energetic particles and the F_2314_ solution in Equation (2) [38].

(1) $$\gamma_{l}(1+\cos\theta )=2{\left(\gamma_{s}^{d}\gamma_{l}^{d}\right)}^{1/2}+2{\left(\gamma_{s}^{p}\gamma_{l}^{p}\right)}^{1/2}$$

(2) $$W_{a}=4\gamma_{s}^{d}\gamma_{l}^{d}/(\gamma_{s}^{d}+\gamma_{l}^{d})+4\gamma_{s}^{p}\gamma_{l}^{p}/(\gamma_{s}^{p}+\gamma_{l}^{p})$$

where $\gamma_{l}$ is the surface energy of liquid; *θ* is the contact angle; $\gamma_{l}^{d}$ (or $\gamma_{s}^{d}$) and $\gamma_{l}^{p}$ (or $\gamma_{s}^{p}$) are the dispersion component and polarity component of liquid (or solid), respectively; *W_a_* is the interfacial adhesion work. The surface energies of the CL-20/HMX cocrystal and CH@MF composites, together with the interfacial adhesion work between the CH/CH@MF composites and F_2314_, are listed in Table 3. The surface energy components $\gamma_{s}^{p}$ and $\gamma_{s}^{d}$ of F_2314_ are known to be 28.52 and 1.15 mN/m [39]. As shown in Table 3, after coating with the MF resin, the value of the surface energy increased with the increasing shell content. In addition, the increased $\gamma^{d}$ and decreased $\gamma^{p}$ narrowed the polar gap between the CH@MF composites and F_2314_, and it facilitated the uniform dispersion of the composite particles in the nonpolar F_2314_ solution. More importantly, the adhesion work (*W_a_*) between the CH@MF composites and F_2314_ gradually enhanced with the addition of MF resin, ascribed to the enriched interfacial interactions such as hydrogen bonding with the –OH groups in MF as proton donors and –F groups in the F_2314_ chains as proton acceptors.

### 2.4. Thermal Properties

**Figure 8:**
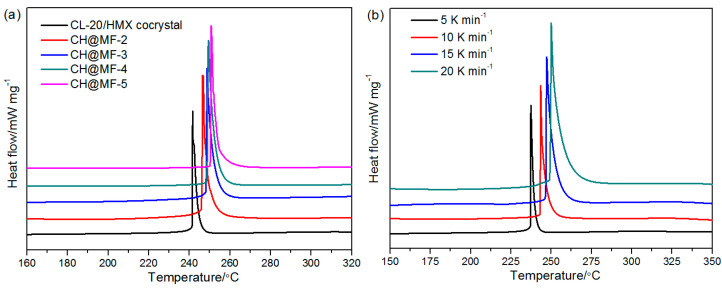

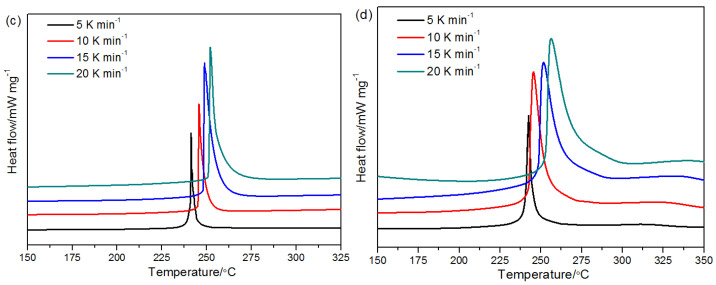
DSC curves of the CL-20/HMX cocrystal and CH@MF composites with a heating rate of 10 °C/min (**a**); DSC curves at different heating rates for CH@MF–2 (**b**), CH@MF–3 (**c**) and CH@MF–4 (**d**).

To detect the thermal properties of the coated composites, the initial and modified samples were studied by DSC as shown in Figure 8. It is clear that the CL-20/HMX cocrystal exhibited one remarkable exothermic peak at 241.2 °C with a heating rate of 10 °C/min. The decomposition peak temperatures of CH@MF–2, CH@MF–3, CH@MF–4 and CH@MF–5 were found to be 246.5, 248.7, 249.5 and 250.8 °C, respectively, indicating that the low content of MF made significant improvements to the thermal stability of the cocrystal. This may be explained from two aspects: one is that the MF resin is a heat-resistant material with only one endothermic peak at 416.1 °C according to the literature [37]; the other is that in situ polymerization endows the coated cocrystal full-scale coverage and strong interfacial interactions, resulting in enhanced thermal stability. Samples incorporated with the MF resin had similar decomposition peak patterns. The changes in the peak temperature demonstrates the possibility and controllability of tuning the thermal stability by adjusting the coating amount of the MF resin.

The kinetics of the CH@MF composites were studied by nonisothermal DSC. Figure 8b–d depict the DSC results of the CH@MF composites with a heating rate of 5, 10, 15 and 20 °C/min, respectively. All three peaks moved right as the heating rate increased. Taking CH@MF–2 as an example, the peak temperatures were 237.3, 246.5, 248.3 and 250.4 °C, corresponding to the increase in the heating rate. The kinetic parameters of the composites were calculated by Kissinger, Friedman isoconversional and combined kinetic methods, and the results are listed in Table 4. Generally, a higher activation energy means more energy is needed to reach the activation energy barrier, resulting in a slower initiation of the disintegration reaction [40]. The activation energy for CH@MF–2 was calculated to be 233.42 kJ mol^−1^, and the activation energy (*E*_a_) increased to 259.49 kJ mol^−1^ after the shell content improved to 1.6%. Similarly, the *E*_a_ value for CH@MF–4 was 278.11 kJ mol^−1^. Besides, the linear correlation coefficients were all approximate to 1, supplying good evidence for the calculation accuracy. The values of *A* also proved that the increase in the loading amount of the MF resin reinforced the thermal stability of the coated composites.

**Figure 9:**
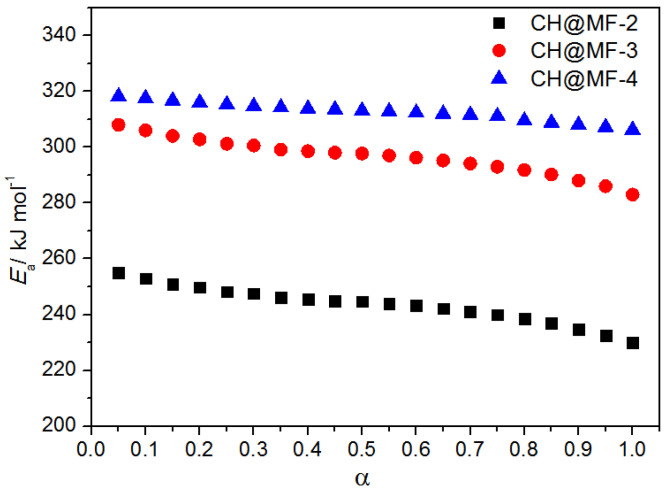
*E*_a_–α relationships for the thermal decomposition processes of the CH@MF composites.

In order to obtain more accurate calculation results, the Fraser–Suzuki equation [41] was adopted to fit the DSC peaks of the original data in the Friedman method. It can be found that all the correlation coefficients were greater than 0.98, which meets the accuracy requirement of the kinetic evaluation. Although the *E*_a_ values obtained by the Friedman method were different from those using the Kissinger method; they still showed a consistent trend of improved activation energy as the shell content increased. The *E*_a_-extent of the conversion (α) relationships of the CH@MF composites are displayed in Figure 9. It is obvious that the *E*_a_ of the CH@MF composites declined gently at the initial stage of the reaction process and then dropped quickly in the scope of α = 0.8~1.0, showing an autocatalytic effect as a result of the aggregation of active reaction sites. The α and reaction rate values of the CH@MF composites as a function of T are plotted in Figure S2.

The activation energies calculated by the combined kinetic method were close to those using the Friedman isoconversional method. Different from these two methods, the Kissinger method only considers the decomposition peak temperature while ignoring the whole decomposition process, and sometimes error exists, especially for those decomposition processes that do not follow the n-order reaction model. Therefore, the kinetic parameters based on the Friedman and combined kinetic methods are considered to be more reliable. The physical models of the decomposition process are described by the parameters *m* and *n*. The obtained models of the CH@MF composites are shown in Figure S3, and the ideal models were taken as references. One can observe that the thermal decomposition of CH@MF–2 followed a three-dimensional growth of the nuclei (A3) model in the range of α = 0~0.56, while that of CH@MF–3 and CH@MF–4 followed a model between two-dimensional growth of the nuclei (A2) and A3. This suggests that the composites experienced nucleation and nucleus growth in the initial stage of the thermal decomposition process. Then, the decomposition models were close to the random chain scission model (L2), implying that the collapse of the cocrystal molecules was the essential rate-limiting decomposition step of the CH@MF composites. The decomposition of nitramine explosives is commonly dominated by the breakage of the N–NO_2_ bonds. As the bond breaking rate was less relied on in the shell layer herein, the first-step decomposition mechanism of the coated composites was very close to that of pristine cocrystal.

### 2.5. Sensitivity and Detonation Performance

The impact and friction sensitivity of the CL-20/HMX cocrystal, the mixture of MF resin and cocrystal, and the CH@MF composites are presented in Table 5. It can be found that the CL-20/HMX cocrystal possessed the same level of sensitivity as HMX with an impact sensitivity (*H*_50_) of 27.1 cm and a friction sensitivity (*P*) of 72%. It was noted that core–shell coating endows a significant desensitization effect to the energetic cocrystals, especially compared to the physical mixed samples. Taking CH@MF–2 composite as an example, the *H*_50_ increased to 40.6 cm and the friction sensitivity decreased to 50%, while the physical mixture (denoted as CH + MF) with the same molar ratio showed an unsatisfactory desensitization effect. It may be originated from the core–shell structure that the corners and edges of the cocrystal were perfectly wrapped by the coating shell in the core–shell composites, while physical mixing could not fulfill this function. This resulted in that the CH@MF composites featured an intact coating shell with few cracks or deformations, preventing the cocrystals from extrusion, shear and friction to reduce the formation of hot spots. Moreover, the tough MF shell could behave as a cushion layer to disperse the abrupt impact or friction energy. The CH@MF–5 composite with a 3.5% shell content exhibited a remarkably reduced sensitivity with an *H*_50_ of 75 cm and *P* of 17%.

**Figure 10:**
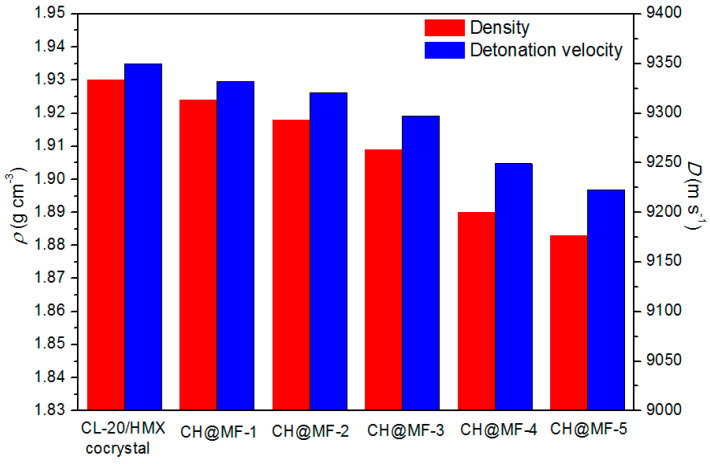
The measured density (ρ) and detonation velocity (D) of the CL-20/HMX cocrystal and CH@MF composites.

Density and detonation velocity are two important parameters reflecting the energetic performance of an energetic material. The densities of the CL-20/HMX cocrystal and CH@MF composites were measured based on the gas expansion displacement method. According to the Urizar equation, the detonation velocity of the composite explosives can be calculated by [25]:

(3) $$\omega_{i}=V_{i}/\Sigma V_{i}$$

(4) $$D=\Sigma w_{i}D_{i}$$

where *V_i_* is the volume of component i; *w_i_* is the volume fraction; *D_i_* is the characteristic detonation velocity. The detonation velocity for the CL-20/HMX cocrystal and MF resin were approximately 9350 and 5400 m/s, respectively. The measured density and detonation velocity of the cocrystal samples are illustrated in Figure 10. One can find that the difference in the densities of all the composites was less than 2.5%, and the detonation velocity loss was lower than 1.5% compared with the raw cocrystal explosive. The impact and friction sensitivity of the CH@MF-3 composite at a 1.6% shell content was almost cut in half with less than 1% energy loss, confirming the superiority of the core–shell structure constructed by in situ polymerization.

Previous work was conducted by our group on the application of CL-20/HMX cocrystal in composite-modified double-base (CMDB) propellants [42,43]. The CMDB propellant containing CL-20/HMX cocrystal exhibited enhanced combustion properties, stability and mechanical properties. However, the stability improvement was limited, as the impact sensitivity of CL-20/HMX cocrystal was almost the same as that of β-HMX. One can speculate that the replacement of the CL-20/HMX cocrystal with CH@MF composites would improve the stability of the CMDB propellant markedly with little influence on the energy performance. In addition, the elevated adhesion work between CH@MF particles and F_2314_ binder highlights their potential applications in PBX formulations.

### 2.6. Proposed Formation Mechanism

**Figure 11:**
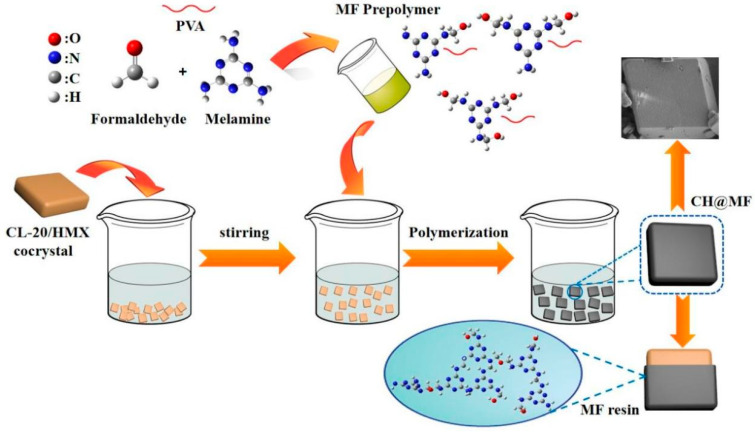
Proposed formation mechanism of the CH@MF composites.

The preparation process and proposed formation mechanism of the CH@MF composites are illustrated in Figure 11. At first, the amine hydrogens of melamine were partially or completely reacted with formaldehyde to provide melamine–formaldehyde prepolymer by an addition reaction. Then, the raw cocrystals were dispersed uniformly in deionized water, and the MF shell was synthesized via in situ polymerization of melamine–formaldehyde prepolymers and deposited on the surface of cocrystal, which occurred under an acidic condition. This process can be illustrated in three steps: Firstly, with the addition of citric acid, MF prepolymers were activated and the degree of cross-linking increased owing to the reduction of the hydroxyl concentration. Secondly, as the pH decreased, the solubility of the MF resin was reduced, and the amino resins gathered around the dispersed cocrystal to create the primary shell. The hydrogen bonding interactions between the amino and hydroxyl groups of the MF resin and nitro groups of the cocrystal played a vital role in the enrichment of resin molecules within the interface. Finally, the polycondensation rate at the interface proceeded much faster than in the water dispersion medium and a tough and firm shell formed eventually. It is worth noting that a small amount of PVA was added in the step of MF prepolymer preparation and performed an important part of the coating process. For one thing, PVA helped improve the surface wettability of the cocrystal explosives and increased the interaction between the energetic cocrystal and the MF resin. Moreover, the PVA served as a supplement to the MF resin to enhance the shell’s flexibility and behaved well in desensitization. The medium in the polymerization process was water, which avoided the trouble of using high solvent and recovery, ensuring the safety of the experiment to the greatest extent and reducing the possibility of pollution and fire. The production cost could be controlled in an acceptable range due to the cheap and accessible raw materials (melamine, formaldehyde solution, etc.) and water reaction medium. Due to the good operability, controllable cost and profitable processing performance, this green, simple and convenient in situ polymerization method is desirable for the desensitization of cocrystal explosives and is promising for scaled-up preparation and industrial application of core–shell composites. 

## 3. Materials and Methods

### 3.1. Materials

The CL-20/HMX cocrystal was prepared by following the previously described self-assembly method with a purity of 98.8% [13]. The melamine, triethanolamine, polyvinyl alcohol (PVA; 88% hydrolyzed, 4.5~6.0 mPa·s), citric acid and formaldehyde solution (37 wt%) were commercially purchased from Aladdin (Shanghai, China), and used without further purification. 

### 3.2. Sample Preparation

The preparation of MF prepolymers was typically fabricated according to the literature [37]. The structure characterizations of MF pre-polymer are shown in Figure S4–S7. Briefly, 1.5 g CL-20/HMX cocrystal was dispersed in 7.5 mL deionized water, and then certain amounts of the MF prepolymer solution were added according to the demanded shell content. The pH value of the mixture was tuned to 4.0~5.0 with 10.0 wt% citric acid solution. Polymerization of the MF prepolymers and coating were attained with continuous agitation for 2 h at 65 °C. Afterwards, the suspension was cooled down to room temperature. Finally, the resultant CH@MF composites were filtered, washed and dried at 60 °C for 12 h. In addition, the physical mixtures of the CL-20/HMX cocrystal and MF resin were prepared with an equal number of components as a contrast. 

### 3.3. Characterization

The morphology of the samples was detected under a scanning electron microscope (SEM; Hitachi S4800) and further characterized by atomic force microscope (AFM; Bruker Dimension Icon) in a tapping mode. Fourier transform infrared spectra (FT-IR) were collected through a NEXUS 870 spectrometer with KBr pellets. Raman spectra were measured under Renishaw inVia instrument with a 785 nm laser. The powder X-ray diffraction (PXRD) patterns were recorded on a Bruker D8 Advance diffractometer under Cu-Kα radiation (λ = 0.154056 nm). The surface chemical composition was measured by performing an X-ray photoelectron spectroscopy (XPS) test on a Thermo Scientific spectrometer. Surface adhesion properties were characterized on a Dataphysics-OCA20 instrument. Thermal behavior was performed with a differential scanning calorimeter (DSC; Netzsch STA 449 F3). The impact sensitivity was tested with a WL-1-type impact sensitivity instrument according to the Chinese GJB-772A-97 standard method 601.2. The friction sensitivity was tested with a WM-1-type friction sensitivity instrument according to the Chinese GJB-772A-97 standard method 602.1. Explosion probability (P) was adopted to assess the friction sensitivity of each sample. The density of the samples was tested under a fully automatic true density instrument (AccuPyc II 1340).

## 4. Conclusions

In this paper, MF-resin-coated CL-20/HMX cocrystals were prepared via a facile in situ polymerization technique. Such a route is green, facile and general for cocrystal explosives, and it was elucidated by a proposed formation mechanism. An intact and uniform MF coating shell was verified by in-depth observation of the morphology and structure. As a result, the cocrystals were successfully coated by low contents of MF resin, and the polymorph of the cocrystal did not change during the whole process. It is inspiring to find that the thermal stability can be effectively enhanced with the thermal decomposition peak temperature increased by 5.3 °C at a 1.0% shell content. The impact and friction sensitivity of the cocrystal explosive could be reduced by more than two times at a 1.6 wt% MF content with less than 1% energy loss, suggesting excellent desensitization effectiveness through in situ coating. With the enhanced thermal and safety performance, CH@MF composites are expected to replace CL-20/HMX cocrystal in CMDB propellant to address the safety concerns. The reinforced interfacial adhesion between the cocrystal composites and fluoropolymer promote their further application in PBX formulations.

## Figures and Tables

**Table 1:** Surface roughness parameters of the CL-20/HMX cocrystal and CH@MF composites.

Samples	R_a_^1^/nm	R_q_^1^/nm	R_max_^1^/nm
CL-20/HMX cocrystal	4.6 ± 0.30	3.3 ± 0.12	73 ± 0.58
CH@MF–2	29.8 ± 0.62	22.7 ± 0.47	331 ± 0.79
CH@MF–3	17.4 ± 0.69	10.1 ± 0.48	213 ± 0.88
CH@MF–4	37.2 ± 0.78	23.9 ± 0.51	474 ± 1.24
CH@MF–5	46.2 ± 0.60	26.4 ± 0.53	591 ± 1.08

^1^ R_a_: mean value of the roughness; R_q_: root mean square of the roughness; R_max_: maximum height value from peak to valley.

**Table 2:** Surface element composition of the MF resin, CL-20/HMX cocrystal and CH@MF composites.

Samples	C 1s/%	N 1s/%	O 1s/%	C/N
MF resin	72.72	19.24	8.04	3.78
CL-20/HMX cocrystal	28.63	38.28	33.09	0.75
CH@MF–2	51.20	31.90	16.90	1.61
CH@MF–3	58.76	27.95	13.29	2.10
CH@MF–4	64.89	24.60	10.51	2.64

**Table 3:** Surface energies of the CL-20/HMX cocrystal, CH@MF composites and F_2314_ together with *W_a_* between CH/CH@MF and F_2314_.

Sample	Surface Energy (mN/m^2^)			Adhesion Work *W_a_* (mN/m^2^)
	*γ^d^*	*γ^p^*	*γ*	
CL-20/HMX cocrystal	31.86	6.18	38.04	64.07
CH@MF–2	37.33	2.93	40.26	67.97
CH@MF–3	38.02	2.67	40.69	68.40
CH@MF–4	38.56	2.48	41.04	68.72
CH@MF–5	39.58	2.10	41.68	69.28
F_2314_	28.52	1.15	29.67	__

**Table 4:** The kinetic parameters for nonisothermal decomposition of the CH@MF composites.

Samples	Kissinger Method			Friedman Method		Combined Kinetic Method		
	*E*_a(1)_	log*A*	*r*	*E*_a(2)_	*r*	*m*	*n*	*E*_a(3)_
CH@MF–2	233.42	17.86	0.9994	245.76	0.9912	0.786	0.968	258.14
CH@MF–3	259.49	20.35	0.9965	297.62	0.9964	0.725	1.100	305.13
CH@MF–4	278.11	22.24	0.9936	313.11	0.9951	0.762	1.230	315.60

*E*_a_: activation energy, in kJ mol^−1^; log*A*: logarithm of the pre-exponential factor, in min^−1^; *r*: correlation coefficient.

**Table 5:** The impact and friction sensitivity of CL-20/HMX cocrystal (CH), the mixture of CL-20/HMX cocrystal and MF resin, and CH@MF composites.

Samples	MF Content/%	Existent Form of MF	H_50_/cm	P/%
CL-20/HMX cocrystal	0	—	27.1	72
CH + MF	1.0	Physical mixture	29.0	69
CH + MF	1.6	Physical mixture	31.2	65
CH@MF–2	1.0	Core–shell	40.6	50
CH@MF–3	1.6	Core–shell	56.4	38
CH@MF–4	2.8	Core–shell	66.2	26
CH@MF–5	3.5	Core–shell	75.0	17

## Supplementary Materials

The following supporting information can be downloaded at: https://www.mdpi.com/article/10.3390/ijms23126710/s1.

## Symbols and Abbreviations

CL-20	2,4,6,8,10,12-Hexanitro-2,4,6,8,10,12-hexaazaisowurtzitane
HMX	1,3,5,7-Tetranitro-1,3,5,7-tetrazacyclooctane
MF	Melamine–formaldehyde
EMs	Energetic materials
TNT	2,4,6-Trinitrotoluene
BTF	Benzotrifuroxan
DNT	2,5-Dinitrotoluene
MTNP	1-Methyl-3,4,5-trinitropyrazole
MDNT	1-Methyl-3,5-dinitro-1,2,4-triazole
CSEs	Core–shell EMs
CH	CL-20/HMX cocrystal
CH@MF	CL-20/HMX cocrystal@MF resin
F_2314_	Fluoropolymer
PBX	Polymer-bonded explosive
PVA	Polyvinyl alcohol
CMDB	Composite–modified double base
*γ*	Surface energy
*W_a_*	Interfacial adhesion work
*E_a_*	Activation energy
α	Extent of conversion
*r*	Correlation coefficient
R_a_	Mean value of roughness
R_q_	Root mean square roughness
R_max_	Maximum height value from peak to valley
*H*_50_	Characteristic height
*P*	Explosion probability
SEM	Scanning electron microscope
AFM	Atomic force microscope
FT-IR	Fourier transform infrared spectra
PXRD	Powder X-ray diffraction
XPS	X-ray photoelectron spectroscopy
DSC	Differential scanning calorimeter

## References

[1] Geetha M., Nair U.R., Sarwade D.B., Gore G.M., Asthana S.N., Singh H. (2003). Studies on CL-20: The most powerful high energy material. J. Therm. Anal. Calorim..

[2] Zhao X.Q., Shi N.C. (1995). Crystal structure of ε-hexanitrohexaazaisowurtzitane. Chin. Sci. Bull..

[3] Liu N., Duan B.H., Lu X.M., Mo H.C., Xu M.H., Zhang Q., Wang B.Z. (2018). Preparation of CL-20/DNDAP cocrystal by a rapid and continuous spray drying method: An alternative to cocrystal formation. CrystEngComm.

[4] Snyder C.J., Chavez D.E., Imler G.H., Byrd E.F., Leonard P.W., Parrish D.A. (2017). Simple and efficient synthesis of explosive cocrystals containing 3,5-dimethylpyrazol-1-yl-substituted-1,2,4,5-tetrazines. Chem. Eur. J..

[5] Zhang J.H., Shreeve J.M. (2016). Time for pairing: Cocrystals as advanced energetic materials. CrystEngComm.

[6] Bolton O., Matzger A.J. (2011). Improved stability and smart-material functionality realized in an energetic cocrystal. Angew. Chem..

[7] Bolton O., Simke L.R., Pagoria P.F., Matzger A.J. (2012). High power explosive with good sensitivity: A 2:1 cocrystal of CL-20:HMX. Cryst. Growth Des..

[8] Yang Z., Li H., Zhou X., Zhang C., Huang H., Li J., Nie F. (2012). Characterization and properties of a novel energetic-energetic cocrystal explosive composed of HNIW and BTF. Cryst. Growth Des..

[9] Liu K., Zhang G., Luan J., Chen Z., Su P., Shu Y. (2016). Crystal structure, spectrum character and explosive property of a new cocrystal CL-20/DNT. J. Mol. Struct..

[10] Anderson S.R., Dubé P., Krawiec M., Salan J.S., Ende D.J.A., Samuels P. (2016). Promising CL-20-based energetic material by cocrystallization. Propellants Explos. Pyrotech..

[11] Ma Q., Jiang T., Chi Y., Chen Y., Wang J., Huang J., Nie F. (2017). A novel multi-nitrogen 2,4,6,8,10,12-hexanitrohexaazaisowurtzitane-based energetic co-crystal with 1-methyl-3,4,5-trinitropyrazole as a donor: Experimental and theoretical investigations of intermolecular interactions. New J. Chem..

[12] Anderson S.R., Am D.J., Ende J.S., Salan P.S. (2015). Preparation of an energetic-energetic cocrystal using resonant acoustic mixing. Propellants Explos. Pyrotech..

[13] Zhang M.H., Tan Y.X., Zhao X., Zhang J.H., Huang S.L., Zhai Z.H., Liu Y., Yang Z. (2020). Seeking a novel energetic co-crystal strategy through the interfacial self-assembly of CL-20 and HMX nanocrystals. CrystEngComm.

[14] An C., Li H., Ye B., Wang J. (2017). Nano-CL-20/HMX cocrystal explosive for significantly reduced mechanical sensitivity. J. Nanomater..

[15] Lian P., Kang C., Zhang Y.X., Chen S., Lai W.P. (2021). Theoretical study on new high energetic density compounds with high power and specific impulse. FirePhysChem.

[16] Huang B., Xue Z., Fu X., Yan Q.L. (2021). Advanced crystalline energetic materials modified by coating/intercalation techniques. Chem. Eng. J..

[17] Zeng C., Yang Z., Zhang J., Li Y., Gong F. (2019). Enhanced interfacial and mechanical properties of PBX composites via surface modification on energetic crystals. Polymers.

[18] He W., Tao B., Yang Z., Yang G., Guo X., Liu P.J., Yan Q.L. (2019). Mussel-inspired polydopamine-directed crystal growth of core-shell n-Al@PDA@CuO metastable intermixed composites. Chem. Eng. J..

[19] Zeng C., Wang J., He G., Huang C., Yang Z., Liu S., Gong F. (2018). Enhanced water resistance and energy performance of core–shell aluminum nanoparticles via in situ grafting of energetic glycidyl azide polymer. J. Mater. Sci..

[20] Niu C., Jin B., Peng R., Shang Y., Liu Q. (2017). Preparation and characterization of insensitive HMX/rGO/G composites via in situ reduction of graphene oxide. RSC Adv..

[21] Lin C., Gong F., Yang Z., Pan L., Liu S., Li J., Guo S. (2018). Bio-inspired fabrication of core@shell structured TATB/polydopamine microparticles via in situ polymerization with tunable mechanical properties. Polym. Test..

[22] Zhang J., Ding L., Yang Z., Liu X. (2017). Mussel-inspired coating of energetic crystals: A compact core–shell structure with highly enhanced thermal stability. Chem. Eng. J..

[23] He G., Liu J., Gong F., Lin C., Yang Z. (2017). Bioinspired mechanical and thermal conductivity reinforcement of highly explosive-filled polymer composites. Compos. Part A.

[24] Lin C., Huang B., Gong F., Yang Z., Guo S. (2019). Core@double-shell structured energetic composites with reduced sensitivity and enhanced mechanical properties. ACS Appl. Mater. Interfaces.

[25] Lin C., Gong F., Yang Z., Zhao X., Li Y., Zeng C., Li J., Guo S. (2019). Core-shell structured HMX@polydopamine energetic microspheres: Synergistically enhanced mechanical, thermal, and safety performances. Polymers.

[26] He G., Yang Z., Pan L., Zhang J., Liu S., Yan Q.L. (2017). Bioinspired Interfacial Reinforcement of Polymer-based Energetic Composites with High Loading of Solid Explosive Crystals. J. Mater. Chem. A.

[27] Ma X., Li Y., Hussain I., Shen R., Yang G., Zhang K. (2020). Core-shell structured nanoenergetic materials: Preparation and fundamental properties. Adv. Mater..

[28] Duan B.H., Li J.K., Mo H.C., Lu X.M., Xu M.H., Wang B.Z., Liu N. (2021). The art of framework construction: Core–shell structured micro-energetic materials. Molecules.

[29] Chen L., Liu J., He W. (2021). Bio-inspired fabrication of energetic crystals@cellulose nanofibers core-shell composites with improved stability and reduced sensitivity. Compos. Commun..

[30] Lin C., Zeng C., Wen Y., Gong F., He G., Li Y., Yang Z., Ding L., Li J., Guo S. (2020). Litchi-like core-Shell HMX@HPW@PDA microparticles for polymer-bonded energetic composites with low sensitivity and high mechanical properties. ACS Appl. Mater. Interfaces.

[31] Jia X., Cao Q., Guo W., Li C., Shen J., Geng X., Wang J., Hou C. (2019). Synthesis, thermolysis, and solid spherical of RDX/PMMA energetic composite materials. J. Mater. Sci..

[32] He W., Liu P.J., He G.Q., Gozin M., Yan Q.L. (2018). Highly reactive metastable intermixed composites (MICs): Preparation and characterization. Adv. Mater..

[33] Li Y., Yang Z., Zhang J., Pan L., Ding L., Tian X., Zheng X., Gong F. (2017). Fabrication and characterization of HMX@TPEE energetic microspheres with reduced sensitivity and superior toughness properties. Compos. Sci. Technol..

[34] Huang B., Hao X., Zhang H., Yang Z., Ma Z., Li H., Nie F., Huang H. (2014). Ultrasonic approach to the synthesis of HMX@TATB core-shell microparticles with improved mechanical sensitivity. Ultrason. Sonochem..

[35] Zhen B.G., Guo X.D., Liu K.W., Li F.S. (2014). In-situ crystallization coating HMX by BAMO-THF copolyether. Chin. J. Explos. Propellants.

[36] Salaün F., Devaux E., Bourbigot S., Rumeau P. (2009). Influence of process parameters on microcapsules loaded with n-hexadecane prepared by in situ polymerization. Chem. Eng. J..

[37] Yang Z., Ding L., Wu P., Liu Y., Nie F., Huang F. (2015). Fabrication of RDX, HMX and CL-20 based microcapsules via in situ polymerization of melamine–formaldehyde resins with reduced sensitivity. Chem. Eng. J..

[38] Owens D.K., Wendt R.C. (1969). Estimation of the surface free energy of polymers. Polym. Sci..

[39] He G., Li X., Bai L., Meng L., Dai Y. (2020). Multilevel core-shell strategies for improving mechanical properties of energetic polymeric composites by the “grafting-from” route. Compos. Part B.

[40] Tan L., Lu X., Liu N., Yan Q. (2021). Further enhancing thermal stability of thermostable energetic derivatives of dibenzotetraazapentene by polydopamine/graphene oxide coating. Appl. Surf. Sci..

[41] Xue Z.H., Zhang X.X., Huang B.B., Xin B., Yan Q.L. (2020). The structural diversity of hybrid qy-HMX crystals with constraint of 2D dopants and the resulted changes in thermal reactivity. Chem. Eng. J..

[42] Wu Z.K., Liu N., Zheng W., Chen J.B., Song X.D., Wang J.N. (2020). Application and properties of CL-20/HMX Cocrystal in composite modified double base propellants. Propellants Explos. Pyrotech..

[43] Wu Z.K., Pei J.F., Song X.D., Liu N., Li J.G., Zhang M. (2020). The catalytic effects of nano-Fe_2_O_3_ and rGO–Fe_2_O_3_ on the thermal decomposition properties of CL-20/HMX cocrystals. New J. Chem..

