# Supplementary material for: Fabrication of CL-20/HMX Cocrystal@Melamine–Formaldehyde Resin Core–Shell Composites Featuring Enhanced Thermal and Safety Performance via In Situ Polymerization
Source: Int J Mol Sci. 2022 Jun 16;23(12):6710. doi: 10.3390/ijms23126710 (PMC9224366; doi:10.3390/ijms23126710)
Supplement: Supplementary file 1 [file ijms-23-06710-s001.zip › ijms-1754091-supplementary.pdf]

Supporting Information

Fabrication of CL-20/HMX cocrystal@Melamine–Formaldehyde resin core-shell composites featuring enhanced thermal and safety performance via *in situ* polymerization

Binghui Duan[a], Xianming Lu[a], Hongchang Mo[a], Bojun Tan[a], Bozhou Wang[a,b,*], Ning Liu[a,b,*]

[a] Xi'an Modern Chemistry Research Institute, Xi'an 710065, People's Republic of China; duanbinghui@126.com

[b] State Key Laboratory of Fluorine & Nitrogen Chemicals, Xi'an 710065, People's Republic of China

* Correspondence: (B.W.) wbz600@163.com; (N.L.) flackliu@sina.com

Table of contents

Table S1 Assignments of main vibrational bands of IR spectra of MF resin and CL-20/HMX cocrystal

Figure S1 Raman spectra of MF resin, CL-20/HMX cocrystal and CH@MF-3 composite with 1.6% shell content

Table S2 Assignments of main vibrational bands of Raman spectra of MF resin and CL-20/HMX cocrystal

Table S3 XPS atomic concentration of functional groups in MF, CL-20/HMX cocrystal and CH@MF composites

Figure S2 The α-T and the reaction rate-T relationships for thermal decomposition of CH@MF composites

Figure S3 A comparison of the kinetic models for thermal decomposition of

CH@MF composites obtained by a combined kinetic analysis method with the ideal models

Figure S4 ^{1}H NMR spectrum of MF pre-polymer

Figure S5 ^{13}C NMR spectrum of MF pre-polymer

Figure S6 Powder X-ray diffraction pattern of MF pre-polymer.

Figure S7 IR spectrum of MF pre-polymer

Table S1. Assignments of main vibrational bands of IR spectra of MF resin and CL-20/HMX cocrystal.

Assignment	CL-20/HMX cocrystal	MF resin
3427		Hydroxyl, imino and amino stretching
3033	C–H stretching	
1602, 1578	Asymmetric stretching of $-NO_2$	
1561, 1491		C–N stretching in the triazine ring
1395, 1335, 1259	N–N stretching, symmetric stretching of $-NO_2$	
1160		Aliphatic C–N vibration
991	Ring stretching	
810		Ring deformation
655	$-NO_2$ out-of-plane deformation	

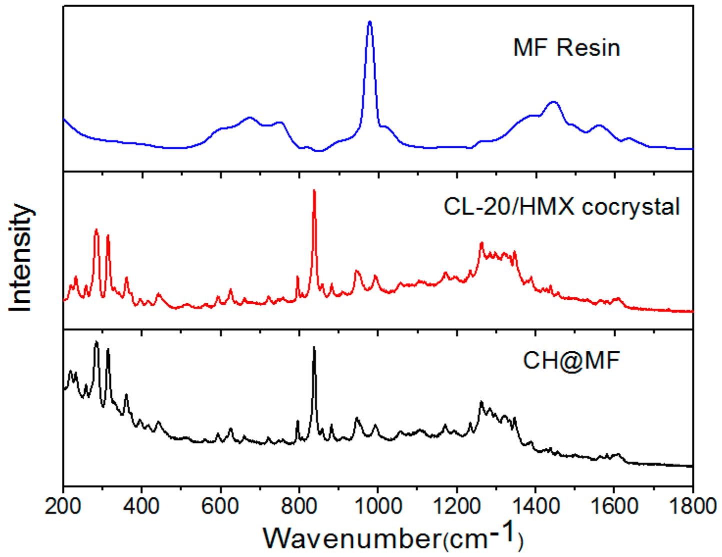

Figure S1. Raman spectra of MF resin, CL-20/HMX cocrystal and CH@MF-3 composite.

Raman spectrum, which is based on inelastic light scattering, is a rapid and nondestructive route for structure characterization. As shown in Figure S1, the obtained characteristic Raman peaks of CL-20/HMX cocrystal were consistent with previous reports. The featured $-NO_2$ asymmetric stretching, symmetric stretching and deformation vibrations were found at position of 1606 cm^{-1}, 1340 cm^{-1} and 834 cm^{-1}, respectively. N–N stretching vibrations were observed at 991 cm^{-1} and 941 cm^{-1}. In addition, the peaks at 280 cm^{-1} and 219 cm^{-1} could be assigned to cage deformation vibration. The characteristic peak positions in

micro-composites are similar to those in raw cocrystal while the intensities vary slightly, indicating the polymorphs of crystals don't change after the coating process and MF resin are successfully coated on the crystals.

Table S2. Assignments of main vibrational bands of Raman spectra of MF resin and CL-20/HMX cocrystal.

Assignment	CL-20/HMX cocrystal	MF resin
1606	Asymmetric stretching of $-NO_2$	
1558, 1444		C–N stretching in the triazine ring
1383, 1340, 1322, 1293	Symmetric stretching of $-NO_2$	
991, 941	N–Nstretching	
977		Aliphatic C–N vibration
834	$-NO_2$deformation	
754, 673		Ring deformation
315	Lattice vibration	
280, 219	Cage deformation	

Table S3 XPS atomic concentration of functional groups in MF resin, CL-20/HMX cocrystal and CH@MF composites.

Atom	Binding energy/eV	Assignment	MF resin	CL-20/HMX cocrystal	CH@MF-2	CH@MF-3	CH@MF-4
C 1s	282.7	<u>C</u>=N	12.12				
	284.9	<u>C</u>–C, <u>C</u>–H, <u>C</u>–N	60.60	6.61	13.55	16.18	15.73
	287.8-288.3	N–<u>C</u>–N		22.02	37.65	42.58	49.16
Sum			72.72	28.63	51.20	58.76	64.89
N 1s	397.8-399.4	C–<u>N</u>	19.24		16.44	23.69	20.50
	401.7-402.1	C–<u>N</u>H–C		20.36	7.57	1.65	1.64
	407.3-407.6	–<u>N</u>O_2		17.92	7.89	2.61	2.46
Sum			19.24	38.28	31.90	27.95	24.60
O 1s	530.8-531.9	C–<u>O</u>–C	8.04		14.20		
	532.9-533.1	C–<u>O</u>–C, –N<u>O</u>$_2$				13.29	10.51
	533.5-533.7	–N<u>O</u>$_2$		33.09	2.70		
Sum			8.04	33.09	16.90	13.29	10.51

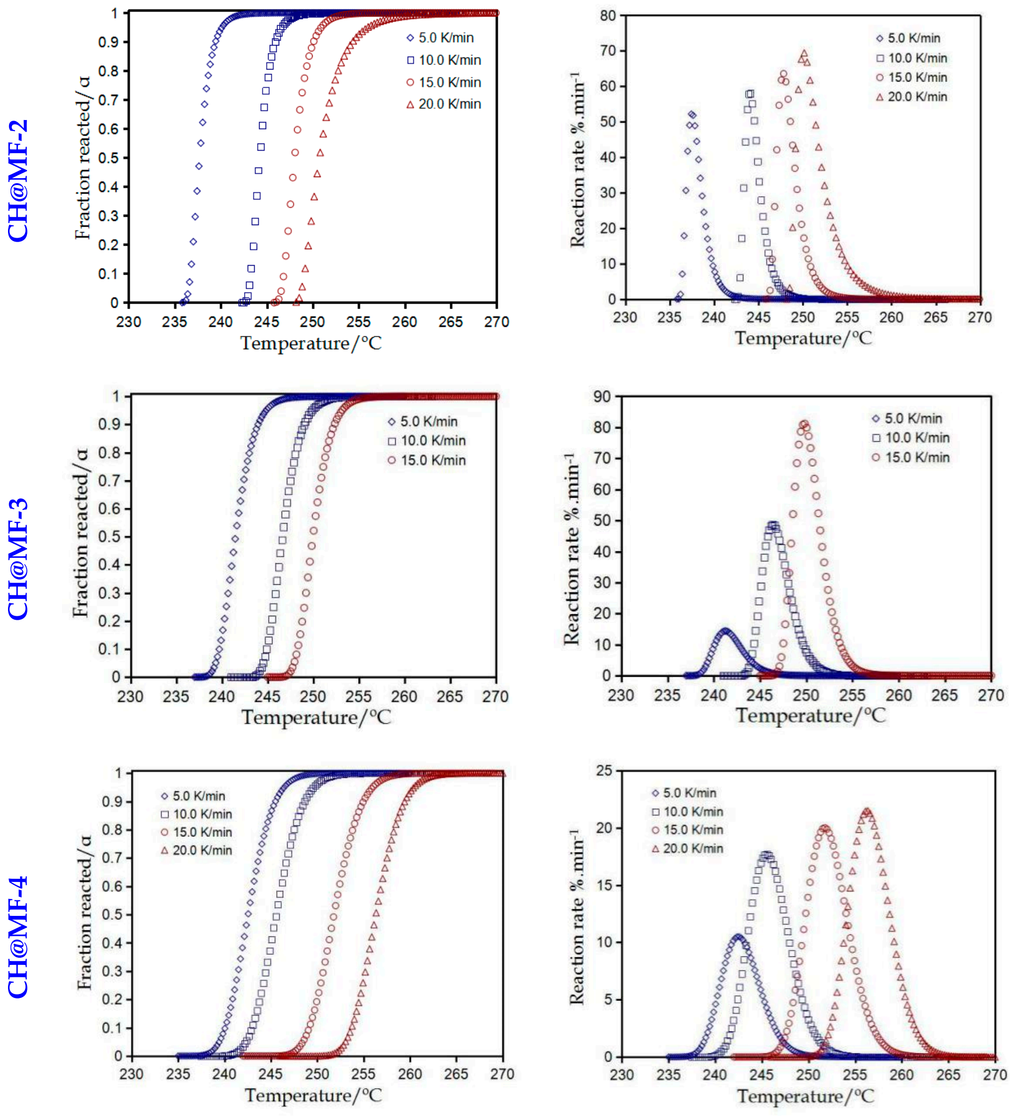

Figure S2. The α-T and the reaction rate-T relationships for thermal decomposition of CH@MF composites.

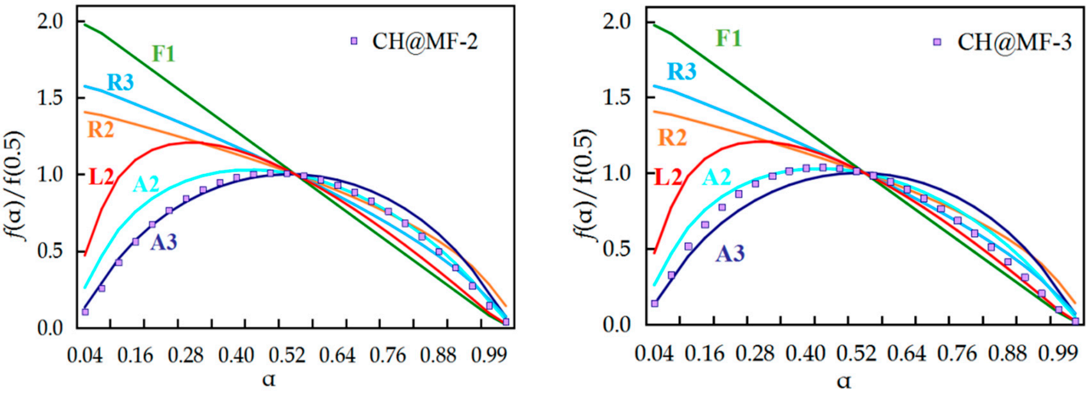

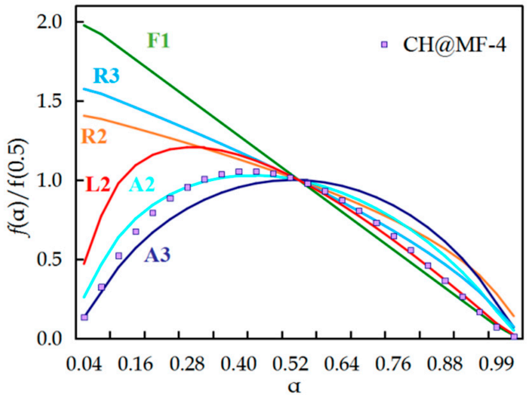

Figure S3. A comparison of the kinetic models for thermal decomposition of CH@MF composites obtained by a combined kinetic analysis method with the ideal models. Notes: F1, First order reaction, so-called unimolecular decay law, where random nucleation followed by an instantaneous growth of nuclei; R2, Phase boundarycontrolled reaction (contracting area); R3, Phase boundary controlled reaction(contracting volume); L2, random chain scission model; A2, A3, Random two and three dimensional nucleation and nucleus growth models.

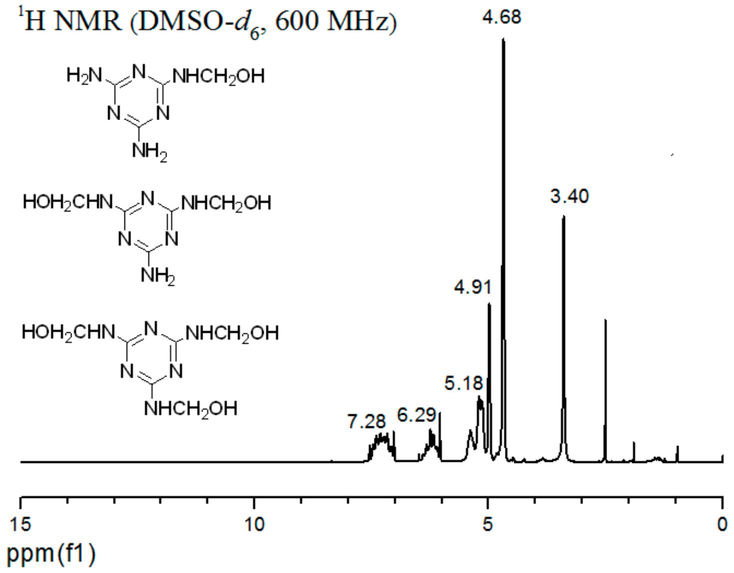

Figure S4. ^{1}H NMR spectrum of MF pre-polymer.

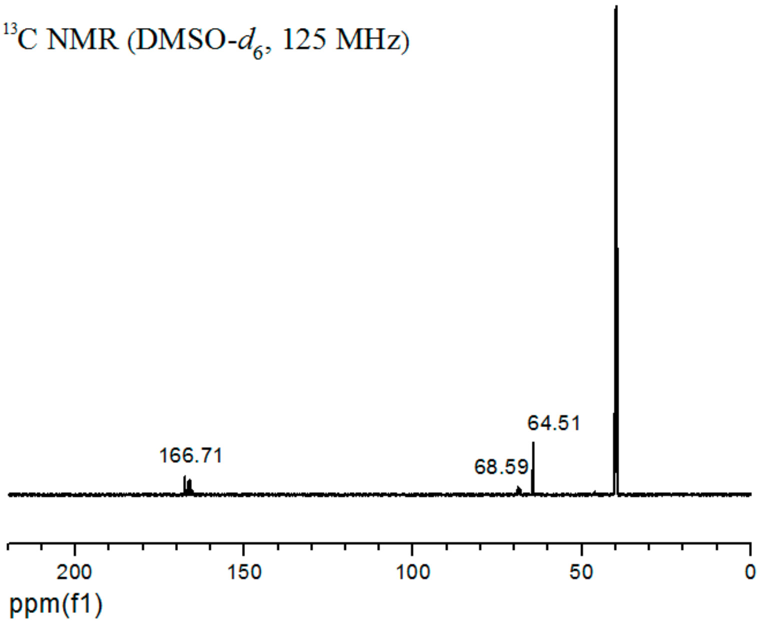

Figure S5. ^{13}C NMR spectrum of MF pre-polymer.

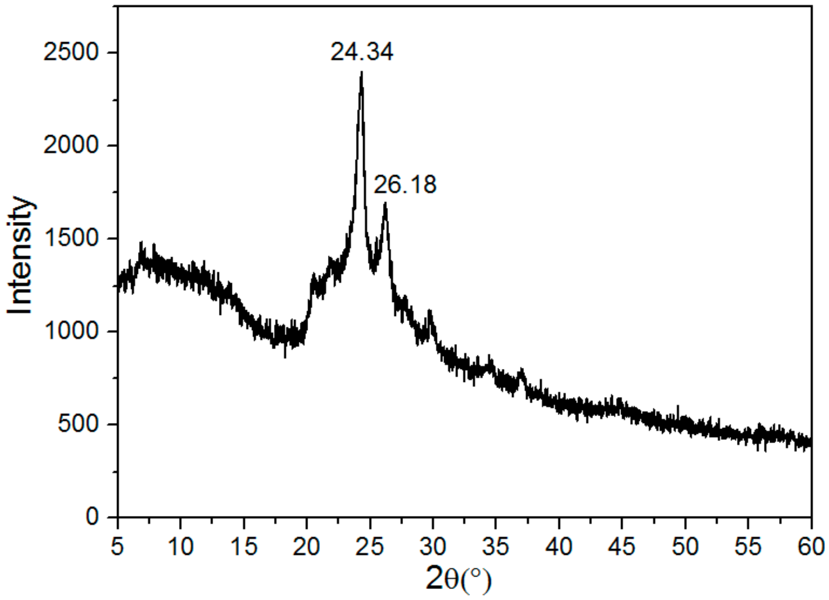

Figure S6. Powder X-ray diffraction pattern of MF pre-polymer.

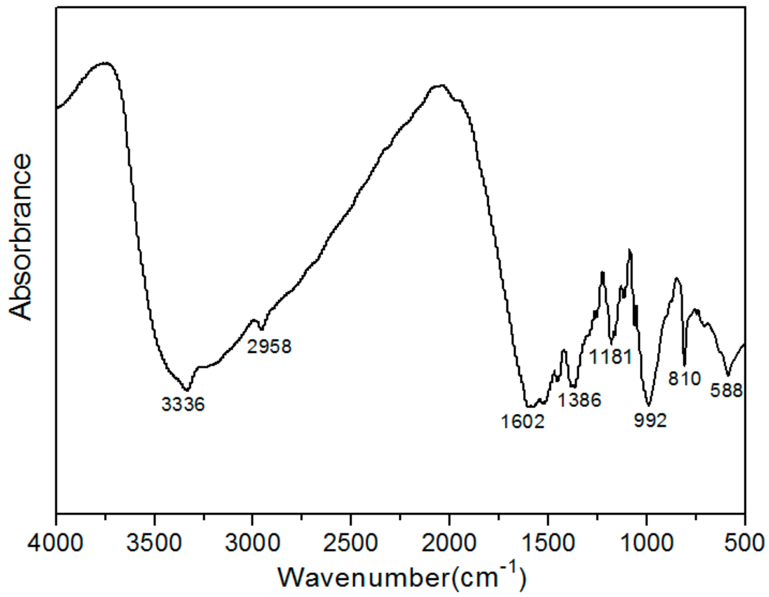

Figure S7. IR spectrum of MF pre-polymer.
